# Orthodontic camouflage versus orthodontic-orthognathic surgical treatment in borderline class III malocclusion: a systematic review

**DOI:** 10.1007/s00784-022-04685-6

**Published:** 2022-09-13

**Authors:** Maged S. Alhammadi, Abeer A. Almashraqi, Ahmed Hassan Khadhi, Khalid Abdullrahman Arishi, Abdelhamid Aidarous Alamir, Essa Mohammed Beleges, Esam Halboub

**Affiliations:** 1 Department of Preventive Dental Sciences, College of Dentistry, Jazan University, Jazan, Saudi Arabia; 2 Department of Orthodontics, Pedodontics and Preventive Dentistry, Faculty of Dentistry, Postgraduate Orthodontic Program, Sana’a University, Sana’a, Yemen; 3 Department of Pre-Clinical Oral Health Sciences, College of Dental Medicine, QU Health, Qatar University, Doha, Qatar; 4 Internship Program, College of Dentistry, Jazan University, Jazan, Saudi Arabia; 5 Department of Maxillofacial Surgery and Diagnostic Sciences, College of Dentistry, Jazan University, Jazan, Saudi Arabia; 6 Department of Oral Medicine, Oral Radiology, and Oral Pathology, Faculty of Dentistry, Sana’a University, Sana’a, Yemen

**Keywords:** Class III malocclusions, Dentoalveolar, Orthodontic camouflage, Orthodontic-orthognathic surgery, Skeletal effect

## Abstract

**Objective:**

This systematic review evaluated the available evidence regarding the skeletal, dentoalveolar, and soft tissue effects of orthodontic camouflage (OC) versus orthodontic-orthognathic surgical (OOS) treatment in borderline class III malocclusion patients.

**Methods:**

**Eligibility criteria**. The included studies were clinical trials and/or follow-up observational studies (retrospective and prospective). **Information sources**. PubMed, Scopus, Science Direct, Web of Science, Cochrane, and LILACS were searched up to October 2021. **Risk of bias**. Downs and Black quality assessment checklist was used. **Synthesis of results**. The outcomes were the skeletal, dentoalveolar, and soft tissue changes obtained from pre- and post-cephalometric measurements.

**Results:**

**Included studies**. Out of 2089 retrieved articles, 6 were eligible and thus included in the subsequent analyses. Their overall risk of bias was moderate. **Outcome results**. The results are presented as pre- and post-treatment values or mean changes in both groups. Two studies reported significant retrusion of the maxillary and mandibular bases in OC, in contrast to significant maxillary protrusion and mandibular retrusion with increased ANB angle in OOS. Regarding the vertical jaw relation, one study reported a significant decrease in mandibular plane inclination in OC and a significant increase in OOS. Most of the included studies reported a significant proclination in the maxillary incisors in both groups. Three studies reported a significant proclination of the mandibular incisors in OOS, while four studies reported retroclination in OC.

**Conclusion:**

**Interpretation**. The OSS has a protrusive effect on the maxillary base, retrusive effect on the mandibular base, and thus improvement in the sagittal relationship accompanied with a clockwise rotational effect on the mandibular plane. The OC has more proclination effect on the maxillary incisors and retroclination effect on the mandibular incisors compared to OOS. **Limitation**. Meta-analysis was not possible due to considerable variations among the included studies. Owing to the fact that some important data in the included studies were missing, conducting further studies with more standardized methodologies is highly urgent. **Registration**. The protocol for this systematic review was registered at the International Prospective Register of Systematic Reviews (PROSPERO, No.: CRD42020199591).

**Clinical relevance:**

The common features including skeletal, dental, and soft tissue characteristics of borderline class III malocclusion cases make it more difficult to select the most appropriate treatment modality that can be either OC or OOS. The availability of high-level evidence—systematic reviews—makes the clinical decision much more clear and based on scientific basis rather than personal preference.

**Supplementary Information:**

The online version contains supplementary material available at 10.1007/s00784-022-04685-6.

## Introduction

### Rationale

Malocclusion is the third most common oral health problem following caries and periodontal diseases [1, 2]. Based on a recent systematic review, the worldwide prevalence of class III malocclusion in the permanent dentition is estimated as low as 0.7% in Israel to as high as 19.9% in China [3]. Although it is less prevalent than other malocclusions traits, it is associated with the greatest facial disfigurement [4]. This type of malocclusion is referred to as a heterogeneous clustering of dentofacial anomalies characterized predominantly by forward positioning of the mandible relative to the maxilla either as an isolated trait or as a part of a syndrome [5].

Treatment of class III malocclusion is age and severity dependent. During childhood, the treatment is age dependent as it can be orthopedically treated through maxillary advancement using reverse traction forces by face mask appliance in the pre-pubertal age. During adulthood, the treatment is severity dependent; mild class III malocclusion with acceptable facial profile can be managed through camouflage: a compensatory orthodontic treatment that involves displacing teeth relative to their supporting bone to mask for an underlying jaw discrepancy [6] with an ultimate aim of attaining acceptable occlusion, esthetics, and function [7, 8] while severe cases with unacceptable facial profile are only amenable to orthognathic surgery, which includes maxillary advancement, mandibular setback, or a combination of both. The aforementioned orthognathic surgery is mostly preceded by conventional orthodontic treatment phase.

Between these two extremes of severity, there are borderline class III malocclusion cases that require special attention and detailed analysis in order to choose any of the above two mentioned therapeutic approaches. The following must be cautiously considered ahead of treatment selection: (1) the extent of facial impairment and its importance to the patient; (2) the anteroposterior position and inclination of maxillary and mandibular incisors; (3) the degree of protrusion of the mandibular symphysis; and (4) the patient acceptance of the selected option [8, 9].

Several studies compared the skeletal and dentoalveolar effects of orthodontic camouflage (OC) versus orthodontic-orthognathic surgical (OOS) treatment of borderline class III malocclusion. Douzartzidis et al. [10] reported more attractive facial profile among OC compared to OOS. On the other hand, Adamian concluded that both treatments resulted in similar/comparable esthetic improvement in profile attractiveness [11].

### Objectives

The aim of this systematic review was to assess the available evidence regarding the skeletal changes and to evaluate the dentoalveolar and soft tissue effects of OC versus OOS treatment in borderline class III malocclusion patients.

## Materials and methods

### Protocol registration

The study protocol was registered at the International Prospective Register of Systematic Reviews (PROSPERO; Registration Number: CRD42020199591) and was conducted according to the guidelines of the Cochrane Handbook for Systematic Reviews of Interventions (http://ohg.cochrane.org).

### PICOS question and eligibility criteria

Table 1 shows the PICOS (Population, Intervention, Comparison, Outcome and Study design) components along with the inclusion and exclusion criteria.

**Table 1:** PICOS components, inclusion and exclusion criteria, and search keywords used for the study selection

Components	Inclusion criteria	Exclusion criteria
Participants	Adult patients with skeletal class III malocclusion	Patients with craniofacial anomalies and/or skeletal asymmetries
	Keyword: Skeletal class III malocclusion, skeletal class III, mandibular prognathism, mandibular protrusion, mandibular hyperplasia, maxillary retrusion, maxillary hypoplasia, maxillary retrognathism, angle class III	
Intervention	Orthognathic	Orthopedic or interceptive or early treatment
	Keyword: Orthognathic surgery, orthognathic surgeries, maxillofacial orthognathic surgery, maxillofacial orthognathic surgeries, orthognathic surgical procedure, orthognathic surgical procedures, mandibular surgery, mandibular surgeries, surgical procedure, surgical procedures, jaw surgery, jaw surgeries	
Comparator	Orthodontic camouflage	Studies with no control group
	Keyword: Orthodontic treatment, camouflage treatment, comprehensive treatment, adult treatment	
Outcome	Primary outcome: skeletal change Secondary outcomes: dentoalveolar and soft tissue changes	Outcomes other than skeletal and dentoalveolar and soft tissue changes
Study design	Longitudinal (retrospective or prospective) studies, and controlled and non-controlled clinical trials	Case reports, case series, literature reviews, systematic review, opinion articles, book chapters

### Information sources, search strategy, and study selection

Two groups of co-authors (two co-authors each) performed an independent comprehensive search in August 2020 in the following six search engines/databases: PubMed, Scopus, ScienceDirect, Web of Science, Cochrane, and LILACS. The search was updated in October 2021, and augmented with a manual search in the reference lists of the included studies. The search keywords of each component of the PICOS question are listed in Table 1.

Two co-authors (M.A. and E.H.) independently screened the retrieved studies for potential inclusion. In brief, duplicates were removed. Then, the titles and abstracts of the remaining articles were screened, and the irrelevant studies were excluded. The full texts of the remaining articles were thoroughly read, and the irrelevant studies were removed. At this stage, the potentiality of the remaining studies to be included was independently assessed by all co-authors. Disagreements, if any, were resolved via consensus. This systematic review was reported according to the Preferred Reporting Items for Systematic Reviews and Meta-Analyses (PRISMA) statement [12].

### Data collection

Data extraction was performed independently by two co-authors (M.A. and A.A.), and disagreements, if any, were discussed with a third co-author (E.H.). The procedure followed a pre-designed template. The following qualitative and quantitative data were extracted: author and year of publication; study design; setting; sample selection criteria and sample size; gender; age of patients; type of surgery; genioplasty; pre-surgical extraction therapy; surgical technique/type of fixation; method of compensation or camouflage; pattern of extraction treatment; method of outcome assessment (2D/3D); and the outcomes measured for assessment of skeletal, dentoalveolar, and soft tissue profile. The treatment changes in (or pre- and post-measurements of) skeletal, dentoalveolar, and soft tissue outcomes obtained from cephalometric measurements were retrieved for each individual group (OC and OOS). The most commonly used measurements describing the following were obtained: maxillary base position (SNA), mandibular base position (SNB), sagittal skeletal relation (ANB), vertical skeletal relation (MPA), maxillary incisor inclination, mandibular incisor inclination, upper lip position, lower lip position, and nasolabial angle.

### Primary outcome

The primary outcome included the following skeletal changes: (1) maxillary skeletal position, (2) mandibular skeletal position, (3) sagittal skeletal jaw relation, (4) vertical skeletal jaw.

### Secondary outcomes

The secondary outcomes were the dentoalveolar and soft tissue changes which could be classified under five categories: (1) maxillary incisor inclination, (2) mandibular incisor inclination, (3) upper lip position/E-line, (4) lower lip position/E-line, and (5) nasolabial angle.

### Risk of bias

The risk of bias was assessed following Downs and Black checklist for assessment of the methodological quality of non-randomized studies [13]. The checklist comprises a total of 27 items distributed between 5 sub-scales with a maximum score of 32 points: 10 items for quality of reporting, 3 items for external validity, 7 items for internal validity in term of bias, 6 items for internal validity in terms of confounding, and 1 item for statistical power. The assessment was done independently by two co-authors (M.A. and E.H.), and disagreements, if any, were resolved via consensus. The studies were categorized as low, medium, and high levels of quality if their scores were ≤ 16, 17–26, and 27–32, respectively.

### Statistical analyses

Only two included studies [14, 15] reported the mean changes (the difference between pre- and post-treatment measurements) of some of the outcomes of interest. However, these two studies applied different protocols of extraction or non-extraction in both OC and OOS groups. The other included studies [16–19] did not report the mean changes. Hence, we sent several emails to the corresponding authors, but no response has been received until the time of writing up this article (supplementary material 1). Owing to the lack of the mean changes of the included outcomes and the substantial heterogeneity among the included studies, meta-analyses were not conducted. Instead, the included studies were analyzed qualitatively.

## Results

### Study selection

**Fig. 1:**
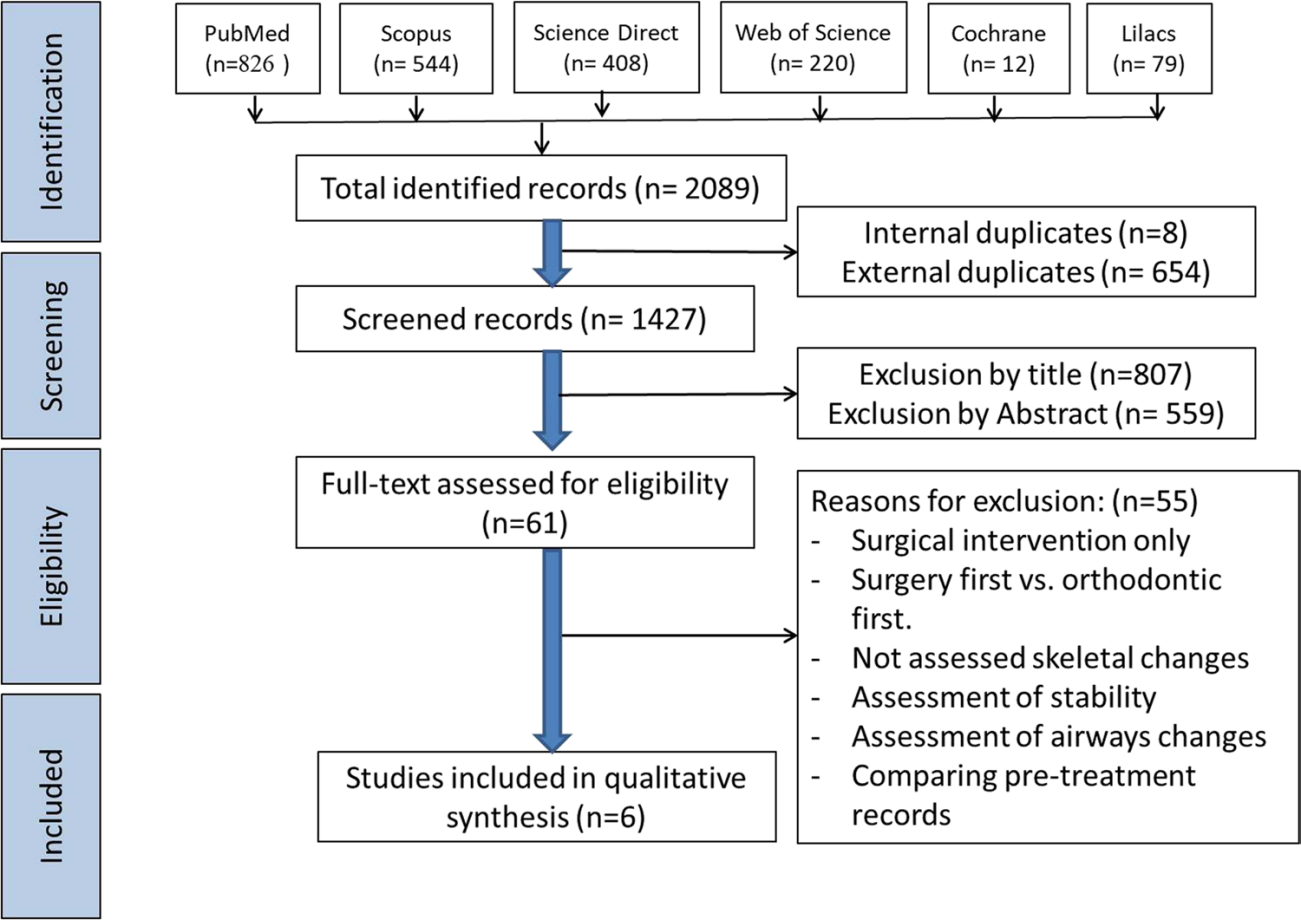
PRISMA diagram of article retrieval

The PRISMA [20] flow chart (Fig. 1) presents the results of the search process. A total of 2089 studies were retrieved, of which 662 were excluded as internal and external duplicates. After screening the remaining 1427 by titles and abstracts, 1366 were excluded due to being irrelevant to the review question. The full texts of the remaining 61 studies were thoroughly read, and 55 were excluded due to different reasons (listed in Fig. 1 and presented in supplementary material 2). The remaining six studies were included in the subsequent analysis.

### Characteristics of the participants and interventions

Table 2 presents the characteristics of the included studies, and details on the procedures and patients’ demographics. All the six studies were of retrospective follow-up design. The inclusion and exclusion criteria were almost the same across the included studies except for Barodiya et al. [16] who selected cases with dental class III although ANB angle was 1 to 4° and the overjet was 1 to 4 mm. In general, the included cases were patients with skeletal class III malocclusion in a non-growing patient with mostly mandibular protrusion, and among the exclusion criteria were noticeable transversal difference, cleft, and/or syndromic diseases.

**Table 2:** Characteristics of included studies

Study	Study type	Population				Intervention: surgical orthodontic treatment			
		Country	Selection criteria	Sample size	Mean age/sex	Type of surgery	Genioplasty (yes/no)	Extractions (yes/no)	Surgical technique/type of fixation
Rabie et al.^17^ 2008	Retrospective	Hong Kong	(1) Southern Chinese, (2) passed the growth spurt, (3) no obvious transversal discrepancy, non-cleft, (4) ANB < 1° for camouflage group and ANB > − 5° for surgical group	*N* = 25 patients Surgery (*N* = 12) Camouflage (*N* = 13)	Surgery 19.4 ± 4.9 years (males = 2, females = 10) Camouflage 16.2 ± 4.9 years (males = 5, females = 8)	Maxillary advancement (1), mandibular setback (2) or bimaxillary surgery (9)	Not available	Not available	Not available
Troy et al.^18^ 2009	Retrospective	USA	(1) White, Black, South Asian, and Asians, (2) passed the growth spurt, (3) overjet of 0 mm or less, (4) unilateral or bilateral angle class III molar relationship, (5) ANB ≤ 0°	N = 72 patients Surgery (*N* = 33) Camouflage (*N* = 39)	Not available	Maxillary advancement, mandibular setback or bimaxillary surgery	Not available	Not available	Rigid fixation
Xiong et al.^23^ 2013	Retrospective	China	(1) adult female and (2) moderate skeletal class III (overjet of − 1 mm to − 4 mm, bilateral angle class III molar relationship, and ANB cephalometric measurement of − 1° to − 4°) No obvious transversal discrepancy, non-cleft, or syndrome	*N* = 46 patients Surgery (*N* = 21) Camouflage (*N* = 25)	Surgery 19.10 ± 2.14 years (females = 21) Camouflage 18.78 ± 3.76 years (females = 25)	Bimaxillary surgery	Not available	Not available	Rigid fixation
Georgalis and Woods ^14^ 2015	Retrospective	Australia	(1) passed the growth spurt, (2) class III molar relationship, (3) incisal overjet ≤ 0 mm, (4) ANB angle less than 1° (5) Wits appraisal less than − 4 mm	*N* = 67 patients Surgery (*N* = 36) Camouflage (*N* = 31)	Not available	Maxillary advancement (3), mandibular setback (2), or bimaxillary surgery (31)	No	14 patients (14, 24) 22 patients (non-extraction)	Not available
Marteniz et al.^15^ 2016	Retrospective	Spain	(1) age over 20 years at the beginning of treatment, (2)Wits appraisal = 3 mm, (3) patients who had not undergone any extractions, (4) patients without any congenital deformity, syndrome, or cleft palate	*N* = 156 patients Surgery (*N* = 79) Camouflage (*N* = 77)	Surgery 23.2 ± 2.6 years (males = 49, females = 30) Camouflage 23.2 ± 2.6 years (males = 41, females = 46)	Maxillary advancement (30), mandibular setback (16) or bimaxillary surgery (33)	Not available	No	Not available
Barodiya et al.^16^ 2021	Retrospective	India	(1) mild skeletal class III (overjet of 1 to 4 mm, (2) bilateral angle class III molar relationship, (3) ANB 1° to 4°, (5) exclusion criteria: noticeable transversal difference, cleft, and/or syndromic diseases	*N* = 46 patients Surgery (*N* = 21) Camouflage (*N* = 25)	Surgery (females = 21) Camouflage (females = 25)	Bimaxillary surgery	Not available	Not available	Rigid fixation

Study	Comparison: orthodontic camouflage treatment		Outcomes			
	Method	Extractions (yes/no)	Method	Skeletal	Dentoalveolar	Soft tissue
Rabie et al.^17^ 2008	Extraction treatment only	8 patients (14, 24, 34, 44) 2 patients (15, 25, 34, 44) 1 patient (13, 23, 34, 44) 1 patient (14, 25, 34, 44) 1 patient (34, 44)	2D	SNA, SNB ANB, MPA	U1 inclination (U1/SN), L1 inclination (L1/MP)	Surgery Holdaway angle (*) Z angle (NS) Camouflage Holdaway angle (NS) Z angle (*)
Troy et al.^18^ 2009	Extraction treatment only	Not available	2D	SNA, SNB ANB	U1 inclination (U1/SN), L1 inclination (L1/MP)	Not available
Xiong et al.^23^ 2013	Not available	Not available	2D	SNA, SNB ANB, MPA	U1 inclination (U1/FH), L1 inclination (L1/MP)	Not available
Georgalis and Woods ^14^ 2015	Extraction or non-extraction treatment only	18 patients (34, 44) 13 patients (non-extraction)	2D	SNA, SNB ANB	U1 inclination (U1/SN), L1 inclination (L1/MP)	Surgery Upper lip-E plane (*) Lower lip-E plane (*) Nasolabial (*) Camouflage Upper lip-E plane (*) Lower lip-E plane (*) Nasolabial (*)
Marteniz et al.^15^ 2016	Non-extraction treatment only	NA	2D	SNA, SNB ANB, MPA	U1 inclination (U1/PP), L1 inclination (L1/MP)	Not available
Barodiya et al.^16^ 2021	Not available	NA	2D	SNA, SNB ANB, MPA	U1 inclination (U1/FH), L1 inclination (L1/MP)	Not available

A total of 412 participants were enrolled in all studies. Two hundred and ten participants were enrolled in the OC, with four studies [15–17, 19] reporting the gender distribution (46 males and 104 females), while the other two studies [14, 18] did not do so. Two hundred and two were enrolled in OOS, with four studies [15–17, 19] reporting the gender distribution (51 males and 82 females), while the other two studies [14, 18] did not do so. Three studies mentioned the mean age of OC ranging from 16.2 ± 4.9 [17] to 23.2 ± 2.6 [15] years, and of OOS ranging from 19.1 ± 2.14 [19] to 23.2 ± 2.6 [15] years. One study [15] calculated the sample size in advance.

In OC, two of the included studies [17, 18] reported extraction treatment, one study [15] reported non-extraction treatment, and one study [14] reported both options, while two studies [16, 19] did not report the camouflaging method. In OOS, two studies [16, 19] reported performing bimaxillary surgery for all participants, while the remaining studies conducted either maxillary advancement, mandibular setback, or bimaxillary surgery. Regarding the extraction treatment in the pre-surgical orthodontic phase, one study [14] reported extraction or non-extraction based on the selected case, one study [15] reported that no extraction was done during this phase, while four studies [16–19] did not report this point. One study [14] reported that genioplasty was not part of the surgical intervention while the other five studies [15–19] did not report this. Three studies [16, 18, 19] mentioned that rigid fixation was performed during the surgery while the other studies [14, 15, 17] did not report the used fixation technique.

As four studies [14, 15, 17, 18] reported the significant values of comparisons between pre- and post-treatment in both studied groups, unlike the other two studies [16, 19] which did not do so, the results of these four studies are presented in the following sections.

### Primary outcome

Regarding the skeletal changes, Table 3 summarizes the results of the anteroposterior and vertical skeletal effects of the OC and OOS. Regarding the anteroposterior plane, two studies reported maxillary base retrusion (based on SNA angle: 0.1 ± 1.2° [14] and 0.29 ± 2.1° [15]) while two studies [17, 18] reported no significant changes in OC. On the other hand, three studies reported significant protrusion of maxillary base (3.4 ± 2.5° [14], 3.20 ± 4.3° [15], and 2.3° [18]) while the fourth one [17] reported no significant effect in OOS. Two studies reported mandibular retrusion (based on ANB angle: 0.2 ± 1.8° [14] and 0.77 ± 1.7° [15]) in OC, while all studies [14, 15, 17, 18] showed significant mandibular retrusion in OOS (from as low as 0.79 [15] to as high as 3.45 [17]).

**Table 3:** Summary of pre- and post-treatment skeletal variables in the camouflage and orthognathic surgery groups in the included studies

Author (year) [reference]	Maxillary base position (SNA°)				Mandibular base position (SNB°)				Sagittal skeletal relation (ANB°)			Vertical skeletal relation (MPA = or SN/Go-Gn°, SN/Go-Me or SN/Ag-Me or FH/Go-Me)		
	Pre-	Post-		Sig	Pre-	Post-		Sig	Pre-	Post-	Sig	Pre-	Post-	Sig
Camouflage														
Rabie et al.^17^ 2008	79.89 ± 2.67	79.11 ± 3.49		NS	81.35 ± 2.81		80.79 ± 2.84	NS	− 1.46 ± 2.06	− 1.68 ± 1.54	NS	33.84 ± 5.23	33.65 ± 6.16	NS
Troy et al.^18^ 2009	80.2 ± 3.4	80.12 ± 3.5		NS	81.63 ± 3.5		81.39 ± 3.5	NS	− 1.43 ± 1.3	− 1.26 ± 1.8	NS	NE	NE	NE
Xiong et al.^23^ 2013	82.24 ± 1.63	82.67 ± 3.24		NR	84.79 ± 1.87		84.24 ± 2.13	NR	− 2.55 ± 1.91	− 1.67 ± 1.23	NR	33.42 ± 4.12	31.56 ± 4.23	NR
Georgalis and Woods ^14^ 2015	80.5 ± 4	80.5 ± 3.9↓		*	81.6 ± 4.1		81.4 ± 4.1↓	*	− 1.2 ± 2	− 0.9 ± 2.2↑	*	NE	NE	NE
Marteniz et al.^15^ 2016	80 ± 4.2	80.3 ± 4.4↓		*	82 ± 4		81.2 ± 4.2↓	*	− 1.9 ± 2.3	− 1 ± 2.8↑	*	33.4 ± 5.9	34.4 ± 6.2↓	*
Barodiya et al.^16^ 2021	82.24 ± 1.63	82.67 ± 3.24		NR	84.79 ± 1.87		84.24 ± 2.13	NR	− 2.55 ± 1.91	− 1.67 ± 1.23	NR	33.42 ± 4.12	31.56 ± 4.23	NR
Orthognathic surgery														
Rabie et al.^17^ 2008	80.96 ± 5.08		80.91 ± 4.63	NS	83.08 ± 6.6		79.62 ± 5.12↓	*	− 2.12 ± 2.51	1.3 ± 2.36↑	*	35.65 ± 6.32	37.01 ± 5.31	NS
Troy et al.^18^ 2009	79.85 ± 3.7		82.08 ± 4.2↑	*	84.33 ± 4.2		82.58 ± 4.4↓	*	− 4.47 ± 2.8	− 0.5 ± 2.4↑	*	NE	NE	NE
Xiong et al.^23^ 2013	82.13 ± 1.12		84.13 ± 3.12	NR	85.25 ± 1.34		82.12 ± 3.09	NR	− 3.12 ± 1.94	2.01 ± 1.34	NR	32.19 ± 3.98	30.28 ± 5.87	NR
Georgalis and Woods ^14^ 2015	79.7 ± 3.2		83.1 ± 3.5↑	*	83.5 ± 3.2		81.6 ± 3.2↓	*	− 3.8 ± 2.4	1.5 ± 2.4↑	*	NE	NE	NE
Marteniz et al.^15^ 2016	80.9 ± 4		84.1 ± 4.2↑	*	84.1 ± 4.2		83.3 ± 3.3↓	*	− 3.2 ± 3.1	0.8 ± 2.5↑	*	34.8 ± 6.6	37 ± 13.9↑	*
Barodiya et al.^16^ 2021	82.13 ± 1.12		84.13 ± 3.12	NR	85.25 ± 1.34		82.12 ± 3.09	NR	− 3.12 ± 1.94	2.01 ± 1.34	NR	32.19 ± 3.98	30.28 ± 5.87	NR

*SNA°*,the angle between 3 point landmarks, S, N, and A point, determining the anteroposterior position of the maxilla relative to the cranial base

*SNB°*, the angle between 3 point landmarks, S, N, and B point, determining the anteroposterior position of the mandible relative to the cranial base

*ANB°*, the angle between 3 point landmarks, A point, N and B point, determining the anteroposterior relation between maxilla and the mandible relative to the cranium

*MPA°*, the angle between the line S–N and the mandibular plane, measuring the mandibular base tipping relative to the cranium

^*^Significant

*NS*, not significant;

*NR*, not reported;

*NE*, not evaluated

Two studies reported a significant increase in the anteroposterior jaw relation (based on ANB angle: 0.3 ± 1.17° [14] and 0.92 ± 1.9° [15]) in OC. This jaw relation was reported to be increased more significantly in OOS: from as low as 3.97° [18] to as high as 5.3° [14]. The vertical jaw relation was evaluated by two studies using two different planes, SN/Go-Me [17] and SN/Ag-Me [15]. One study [17] recorded insignificant change of the mandibular plan angle (MPA) in OC and OOS, while the other study reported a statistically significant decrease in OC by 0.96 ± 1.6°, but significant increase in OOS by 2.17 ± 12.3° [15].

### Secondary outcomes

For dentoalveolar changes, Table 4 shows the results of the treatment effect on the maxillary and mandibular incisor inclination. Three studies [14, 17, 18] evaluated the maxillary incisor inclination relative to the SN plan, while Martinez et al. [15] used the palatal plane as a reference. The proclination effect of OC ranged from as low as 5.31° [18] to as high as 5.6° [14], while it ranged from as low as 4.2 ± 6.7° [15] to as high as 7.6 ± 7.4° [14] in OOS.

**Table 4:** Summary of pre- and post-treatment dentoalveolar and soft tissue variables in the camouflage and orthognathic surgery groups in the included studies

Author (year) [reference]	Maxillary incisor inclination (U1/PP Or U1/SN °Or U1/FH)			Mandibular incisor inclination (IMPA°)			[Upper lip (UL) position/E-line, lower lip (LL) position/E-line and nasolabial angle (NLA)]			
	Pre-	Post-	Sig	Pre-	Post-	Sig	Soft tissue variables	Pre-	Post-	Sig
Camouflage							Georgalis and Woods ^14^ 2015			
Rabie et al.^17^ 2008	111.76 ± 6.02	110.21 ± 4.88	NS	93.74 ± 7.3	86.65 ± 6.59↓	*	UL/E-line	− 5.0 ± 2.6	− 5.3 ± 2.5	*
Troy et al.^18^ 2009	104.96 ± 6.7	110.27 ± 6.1↑	*	91.07 ± 6.8	86.17 ± 7.8↓	*				
Xiong et al.^23^ 2013	71.01 ± 4.83	60.12 ± 7.12	NR	80.18 ± 4.29	73.29 ± 8.23	NR	LL/E-line	− 1.3 ± 3.1	− 2.4 ± 2.7	*
Georgalis and Woods ^14^ 2015	107.2 ± 6.7	112.8 ± 7.6↑	*	84.3 ± 6.8	82.7 ± 5.7↓	*				
Marteniz et al.^15^ 2016	114 ± 5.5	116.7 ± 9.3	NS	86.2 ± 6	79.6 ± 8.1↓	*	NLA	110 ± 9.6	108.7 ± 9.1	*
Barodiya et al.^16^ 2021	71.01 ± 4.83	60.12 ± 7.12	NR	80.18 ± 4.29	73.29 ± 8.23	NR				
Orthognathic surgery							Georgalis and Woods ^14^ 2015			
Rabie et al.^17^ 2008	108.74 ± 11.07	107.28 ± 8.23	NS	86.91 ± 10.97	94.02 ± 7.96↑	*	UL/E-line	− 6.6 ± 3.4	− 3.4 ± 3.7	*
Troy et al.^18^ 2009	108.87 ± 8.4	111.99 ± 9.5↑	*	83.5 ± 8	86.03 ± 7	NS				
Xiong et al.^23^ 2013	72.09 ± 5.72	70.13 ± 6.92	NR	81.56 ± 6.12	88.75 ± 6.34	NR	LL/E-line	− 1.2 ± 4.2	− 1.0 ± 3.7	*
Georgalis and Woods ^14^ 2015	109 ± 8	111.1 ± 7.7↑	*	79.8 ± 8.3	87.4 ± 6.5↑	*				
Marteniz et al.^15^ 2016	112.7 ± 5.5	116.9 ± 7.6↑	*	77.5 ± 8.7	85.4 ± 11.6↑	*	NLA	104 ± 14.0	108.7 ± 13.4	*
Barodiya et al.^16^ 2021	72.09 ± 5.72	70.13 ± 6.92	NR	81.56 ± 6.12	88.75 ± 6.34	NR				

*U1/PP°*. the angle formed between the palatal plane and the long axis of the most protruded maxillary incisor

*U1/SN°*, the angle formed between the cranial base plane and the long axis of the most protruded maxillary incisor

*U1/FH*, the angle formed between the Frankfort horizontal plane and the long axis of the most protruded maxillary incisor

*IMPA°*, the angle formed between the mandibular plane and the long axis of the most protruded mandibular incisor

^*^Significant

*NS*, not significant;

*NR* not reported

The inclination of the mandibular incisors was measured relative to the mandibular plan in all included studies. The treatment effect on the inclination of the mandibular incisors was significant retroclination in OC ranging from as low as 1.4° [14] to as high as 7.09° [17], while it was significant proclination in OOS by 7.11° [17], 7.6° [14], and 7.9° [15].

Regarding soft tissue parameters, two studies [14, 17] reported the soft tissue outcomes, but using different parameters. While Rabie et al. [17] measured Holdaway and Z angles, Georgalis and Woods [14] measured the upper and lower lip positions relative to the E-line and the nasolabial angle. The former study [17] reported significant increase in the Z angle but insignificant changes in the Holdaway angle in OC; opposite findings were reported in OOS. The latter study reported significant improvement in the upper lip position (0.3 ± 1.6 mm), lower lip position (− 1.2 ± 1.7 mm), and nasolabial angle (− 1.3 ± 8°) in OC, and significant changes in the upper lip position (3.3 ± 1.5 mm), lower lip position (0.2 ± 1.5 mm), and nasolabial angle (4.7 ± 9.3°) in the OOS [14].

### Quality assessment

Table 5 presents the summary scores of the risk of bias based on the Downs and Black checklist. All studies showed a moderate overall risk of bias. Most of the shortcomings were attributed to the lack of external validity and the power of the study. The substantial heterogeneity among the included studies precluded conducting meta-analyses. Detailed scores of the individual items for the risk of bias of the Downs and Black checklist are presented in supplementary material 3.

**Table 5:** Risk of bias based on the Downs and Black checklist (full details presented in the supplementary material)

Study	Reporting (Q1–Q10)	External validity (Q11–Q13)	Internal validity—Bias (Q14–Q20)	Internal validity—Confounding (selection bias) (Q21–Q26)	Power (Q27)	Final score
Rabie et al.^**17**^ 2008	7	0	6	6	0	19
Troy et al.^**18**^ 2009	9	0	7	4	2	22
Xiong et al.^**23**^ 2013	7	0	8	4	0	19
Georgalis and Woods ^**14**^ 2015	11	0	7	6	0	24
Marteniz et al.^**15**^ 2016	11	0	8	4	2	25
Barodiya et al.^**16**^ 2021	7	0	8	4	0	19

## Discussion

Orthodontic diagnosis and treatment planning of class III malocclusion is critical and depends on several factors. Severity is the key among these factors, and it ranges from mild dentoalveolar to severe skeletal problems. Generally, OC by dentoalveolar compensation is recommended for milder discrepancies, while OOS is recommended to non-growing patients with more serious dentoalveolar and/or skeletal discrepancies. “Borderline cases” is a term that refers to cases with mild to moderate skeletal discrepancy that can be treated by either OC or OOS. The decision, however, is quite difficult, and largely depends on the benefit-to-risk ratio. The selection of the cases to suit one of these two modalities is mostly based on clinical examination and cephalometric analysis of sagittal and vertical skeletal parameters, degree of dentoalveolar compensation, and facial esthetics; the patient’s preference is not to be overlooked.

### Primary outcome

This systematic review revealed that OC has a protrusive effect on the maxilla. Although some of these studies reported such a protrusion to be statistically significant, it is not so from a clinical point of view. Anyhow, this can be ascribed to the effect of the dentoalveolar protrusion aided by proclination of the maxillary anterior teeth. Al-Nimria et al. [21] claimed that the position of point A is affected by local bone remodeling associated with proclination of the upper incisor in class II division 2 malocclusions. The effect in OOS was exclusively protrusive and of clinical significance, ranging from 2.3 [18] to 3.4° [14]; this is logical with bimaxillary (or maxillary advancement) surgical modalities which entails advancing the maxilla. Overall, OSS has clinically protrusive effect on the maxillary base compared to clinically insignificant effect of OC.

Regarding the treatment effect on the mandibular base, only two of the included studies [14, 15] showed that OC had statistically significant retrusive effect in OC, although that effect cannot be considered clinically significant. This can be attributed to the border line change in the point B position in the horizontal direction following mandibular incisor retraction either by extraction or non-extraction therapy. Al-Abdwani et al. [22] evaluated changes in the cephalometric position of point B due to mandibular incisor inclination caused by orthodontic treatment and reported that each 10° change in the mandibular incisor inclination results in a borderline average change in point B of 0.3 mm in the horizontal plane. Contrastingly, the effect on the mandibular base was totally retrusive in all included studies in OOS, with marked clinical significance; this is clearly due to the mandibular setback component of the bimaxillary surgery. Overall, OOS has more clinically retrusive effect on the mandibular base compared to clinically insignificant effect by OC.

The collective effect of maxillary base protrusion and mandibular base retrusion, either due to the changes in the incisor inclination or due to the basal surgical procedures, results in reducing effect in the sagittal skeletal discrepancy in both studied groups. This change was clinically insignificant in the OC, but statistically and clinically significant in OOS (between 3.97 [18] and 5.3° [14]).

Regarding the vertical jaw relation, it is worthy to mention that only two studies [15, 17] reported a statistically significant increase in the mandibular plan angle in OC. More significant increase was reported by all included studies in OOS. Overall, OOS has more clinically clockwise rotational effect compared to clinically insignificant effect of OC.

### Secondary outcomes

The present systematic review shows that OC had a proclination effect on the maxillary incisors, which was seen more prominently and of a clinical significance in Troy et al. (5.31°) [18] and Georgalis and Woods (5.6°) [14] studies, in contrast to Rabie et al. [17] and Marteniz et al. [15] who reported no change. Such a change is normal compensation effect of the skeletal discrepancy. Similar proclination effect was reported by three studies [14, 15, 18] in OOS, although it is expected to be a retroclination effect as a mean of decompensation in the orthodontic phase prior to the surgical phase. This indicates that the decompensation phase in these studies was based on non-extraction treatment. As presented in Table 2, Martinez et al. [15] prepared the OOS cases by non-extraction protocol, and most of OOS cases in Georgalis and Woods [14] study followed the same protocol, while Troy et al. [18] did not report this. Overall, OC has more clinically proclination effect on the maxillary incisors compared to the clinically insignificant effect by OOS.

Inclination of the mandibular incisors is a critical factor during either the compensation treatment of camouflaged cases or decompensation phase of orthognathic surgical cases. Alhammadi [23] reported a significant correlation between the degree of the mandibular incisor inclination and the sagittal jaw relation; class III malocclusion showed the highest correlation (*r* = 0.346). In the current systematic review, all the included studies [14, 15, 17, 18] showed a significant retroclination of the mandibular incisors in OC, while three studies [15, 17, 18] reported a proclination effect in OOS. Indeed, this is the main play zone in either the camouflage or the surgical therapy. Overall, OC has a clinically significant retroclination effect on the mandibular incisors, while OOS has a clinically proclination effect.

The soft tissue outcomes of interest were not reported well in most of the included studies. Only one study reported the changes in the upper and lower lips and the nasolabial angle. Clinically, the effect was protrusive on the upper lip and retrusive on the lower lip by OOS and OC, respectively. All the other changes were not of clinical value.

Basically, the most important factor that aids to decide which treatment modality (either OC or OOS) is more effective in treatment of borderline class III is the changes in the soft tissue profile, even from patients’ point of view. Unfortunately, this decisive outcome was reported by only one study. Accordingly, it is highly recommended to include these outcomes in any future studies.

### Limitations

In addition to the small number of studies, the overall quality of the included studies was moderate. Indeed, the more the number of the included clinical trials, the less the risk of bias. Pooling together the prospective and retrospective studies was one serious limitation. The invasive nature of orthognathic surgery, when discussed with the patients in context of research project, directs them to choose the less invasive procedures. Hence, many researchers resort to pool the already recoded data on such invasive procedures for better understanding of their effects. Further limitation was that meta-analysis was not possible due to considerable variations among the included studies with regard to study design, main outcomes, included cases, and applied protocol. Another limitation was that we used Downs-Black checklist to assess the risk of bias. Although this checklist was used in many similar reviews, it is recommended for any future research to use Cochrane RoB (for RCT) or ROBINS-I (for NCT) assessment tools: the more relevant and updated tools in this regard. Only English studies were included, and this was another limitation. Hence, the reported treatment effects should be interpreted with caution. Moreover, standardization of gender and participants’ characteristics; pattern of extraction in both groups; and comprehensive outcomes assessment including detailed soft tissue analysis are advised for future studies.

## Conclusions

Keeping in mind the limitations of this review and the moderate quality of the included studies, the following can be concluded:

1. OSS has a clinically significant protrusive effect on the maxillary base accompanied with a clinically significant retrusive effect on the mandibular base compared to the clinically insignificant effect by OC on the same.

2. OSS has a clinically significant improvement in the sagittal relationship accompanied with clockwise rotational effect on the mandibular plan compared to the clinically insignificant effect by OC on the same.

3. OC has a clinically significant proclination effect on the maxillary incisors and a clinically significant retroclination effect on the mandibular incisors compared to the clinically significant proclination effect in OOS.

## Supplementary Information

Below is the link to the electronic supplementary material.

Supplementary file1 (XLSX 10 KB)

Supplementary file2 (XLSX 138 KB)

Supplementary file3 (XLSX 12 KB)
